# A new species of *Laophontella* Thompson IC & Scott A, 1903 (Copepoda, Harpacticoida, Tetragonicipitidae) from Korea, with notes on copepodid V stage

**DOI:** 10.3897/zookeys.1281.192788

**Published:** 2026-06-09

**Authors:** Jihye Seo, Kyuhee Cho, Jimin Lee

**Affiliations:** 1 Ocean Climate Response & Ecosystem Research Department, Korea Institute of Ocean Science & Technology, Busan 49111, Korea Department of Convergence Study on the Ocean Science and Technology, Ocean Science and Technology School, National Korea Maritime & Ocean University Busan Republic of Korea; 2 Department of Convergence Study on the Ocean Science and Technology, Ocean Science and Technology School, National Korea Maritime & Ocean University, Busan 49112, Korea Ocean Climate Response & Ecosystem Research Department, Korea Institute of Ocean Science & Technology Busan Republic of Korea

**Keywords:** Copepodid V stage, interstitial meiofauna, *Laophontella changi* sp. nov., SEM, taxonomy

## Abstract

A new species of the benthic harpacticoid genus *Laophontella* Thompson IC & Scott A, 1903 (Tetragonicipitidae), collected from intertidal habitats along the coast of Korea, is described. *Laophontella changi***sp. nov**. is clearly distinguished from its congeners by the following combination of characters: a seven-segmented female antennule, a ventral cuticular expansion with an undulating edge on the first free abdominal somite of females, a C-shaped proximal dorsolateral expansion and auriform lateral elevation on the caudal rami of females, a markedly elongate inner seta on P4exp-2, and a hoe-shaped outer spine on the P3 endopod of males. The copepodid V stage is also described, allowing assessment of developmental changes and the retention of morphological characters. Detailed morphological examination reveals previously overlooked characters with potential diagnostic value at both the genus and species levels.

## Introduction

The genus *Laophontella* Thompson IC & Scott A, 1903 is a small but morphologically distinctive group within the family Tetragonicipitidae Lang, 1944. The genus is characterized by a subcylindrical habitus, well-developed posterolateral processes on the prosomal somites, a strongly reduced P4 endopod, and a foliaceous female P5 (Por 1964). The genus currently comprises three valid species and three subspecies: the type species by original designation, *Laophontella typica* Thompson IC & Scott A, 1903, and two polytypic species, *Laophontella armata* (Willey, 1935) and *Laophontella horrida* (Por, 1964) (Walter and Boxshall 2026). *Laophontella armata* includes two subspecies, *L. armata armata* and *L. armata indica* Sewell, 1940, whereas *L. horrida* comprises three subspecies: *L. horrida horrida*, *L. horrida dentata* Mielke, 1992, and *L. horrida namibiensis* Kunz, 1994.

Although *Laophontella* includes a relatively small number of species, its taxonomic placement has long been regarded as uncertain due to unclear illustrations and insufficient descriptions of some species, including the type species *L. typica*, which was based on a copepodid-stage specimen. The taxonomic position of *Laophontella* has undergone several revisions since the original description of the genus. *Laophontella typica* was initially described without assignment to a family and later placed in Cletodidae Scott T, 1904 by Monard (1927). Sewell (1940) subsequently expanded the genus by transferring *Phyllopodopsyllus armatus* Willey, 1935 in the family Canthocamptidae Brady, 1880 to *Laophontella* and proposing the subspecies *L. armata indica*. Por (1964) later erected the genus *Willeyella* Por, 1964 within Tetragonicipitidae to accommodate *W. horrida*, and transferred *P. armatus* to this new genus. The current concept of *Laophontella* was stabilized by Lang (1965), who placed the genus in Tetragonicipitidae and synonymized *Willeyella* with it.

Species of *Laophontella* have been recorded from various oceanic regions, including the Atlantic, Pacific, and Indian oceans, as well as the Mediterranean Sea, typically occurring in tropical to subtropical coastal environments. *Laophontella typica* has been documented in Sri Lanka (Thompson and Scott 1903). Subspecies of *L. armata* have been reported from the Bahamas (Geddes 1968), Bermuda (Willey 1935), Andaman-Nicobar Islands, India (Wells and Rao 1987), the Maldives and Nicobar Islands (Sewell 1940), and Inhaca Island, Mozambique (Wells 1967). Subspecies of *L. horrida* have been recorded from several countries, including Nohoch Nah Chich system, Mexico (Gómez and Morales-Serna 2015), Costa Rica (Mielke 1992), Curaçao (Por 1984), São Sebastião Island, Brazil (Björnberg and Kihara 2013), Marseille and Banyuls-sur-Mer, France (Bodin 1964; Guille and Soyer 1966; Bodiou and Soyer 1973), Namibia (Kunz 1994), Israel (Por 1964), Andaman-Nicobar Islands, India (Wells and Rao 1987), and South Korea (Karanovic 2019). In Korean waters, however, Karanovic (2019) provided an incomplete description and several SEM images of *L. horrida dentata*.

During a survey of intertidal meiofauna along the Korean coast, a new species of *Laophontella* was discovered. Both male and female copepodid-stage specimens were also obtained, allowing comparison of morphological changes in key characters between copepodid and adult stages. Herein, we present a detailed description of the new species, supported by scanning electron micrographs.

## Materials and methods

**Figure 1.**
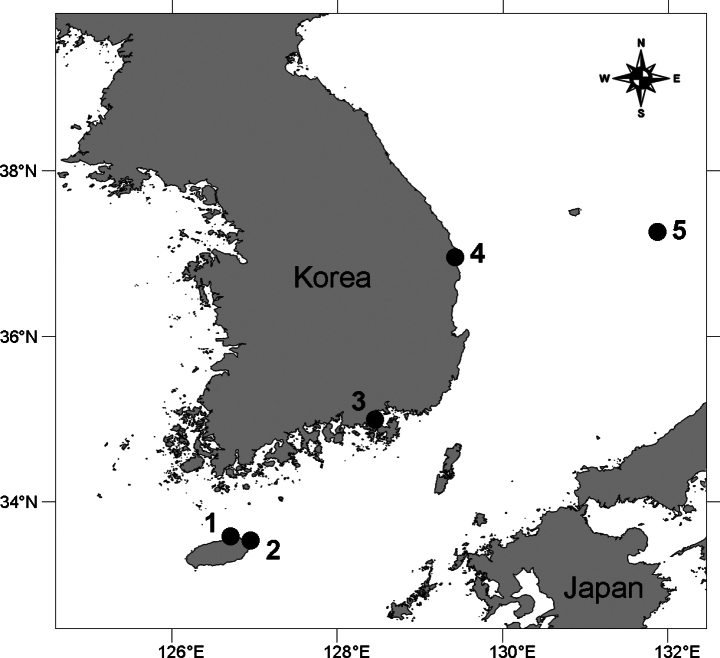
Sampling localities. **1**. Gwangot, Jeju Island; **2**. Deuksaenggot, Udo Islet, Jeju Island; **3**. Janghang port, Goseong; **4**. Daenari port, Uljin; **5**. Mulgol, Dokdo Island.

Sediment samples were collected from intertidal coastal areas along the Korean coast (Fig. 1) using a suction device. Collected materials were anesthetized via exposure to 7.5% magnesium chloride (MgCl_2_) for 30–60 min, sieved through a hand net with a 64 µm mesh, and immediately fixed in 10% formalin. In the laboratory, benthic organisms were separated using the Ludox centrifugation method (Burgess 2001). Harpacticoid copepods were sorted under a stereomicroscope (M165 C; Leica Microsystems, Wetzlar, Germany) and preserved in 5% formalin.

For light-microscopy observation, specimens were dissected in lactic acid and temporarily mounted on hole slides for observation and drawing. After examination, specimens were permanently mounted on H-S slides (double slide plate, BSDS-011R; Biosolution, Daegu, Korea) in lactophenol:glycerin (1:5) (cf. Shirayama et al. 1993). Additional specimens were preserved in vials with 80% ethanol. All drawings were made using a differential interference contrast light microscope (BX53; Olympus, Tokyo, Japan) equipped with a drawing tube.

For scanning electron microscopy, specimens of both sexes were dehydrated through a graded ethanol series of 50–100% at 10% intervals for 30 min each, immersed in hexamethyldisilazane (HMDS), and left to dry completely. The dried specimens were mounted on scanning electron microscopy stubs, coated with carbon using an ion sputter coater (MC1000; Hitachi High-Tech, Tokyo, Japan), and observed using both a conventional scanning electron microscope (SEM) (S-4300; Hitachi, Tokyo, Japan) and a tabletop SEM (SNE-Alpha; SEC Co., Ltd., Suwon, Korea).

Morphological terminology follows Huys et al. (1996). The following abbreviations are used in the text and figures: **ae**, aesthetasc; **CI–CV**, copepodid I–V; **exp**, exopod; **enp**, endopod; **exp(enp)-1(-2, -3)** to denote the proximal (middle, distal) segment of a ramus; **P1–P6**, first to sixth thoracopods. Specimens are deposited in the National Marine Biodiversity Institute of Korea (**MABIK**), Seocheon, Korea, and the Marine Interstitial Fauna Resources Bank (**MInRB**), Korea Institute of Ocean Science & Technology (KIOST), Busan, Korea.

## Results

**Order Harpacticoida Sars, 1903**

**Family Tetragonicipitidae Lang, 1944**

**Genus *Laophontella* Thompson IC & Scott A, 1903**

### Laophontella changi sp. nov.

Taxon classificationAnimaliaHarpacticoidaTetragonicipitidae

B98B7AD1-4D05-5401-9543-BA444B4613B7

https://zoobank.org/B861FC33-4DDC-4C08-A3C9-9A80F09A7903

Figs 2, 3, 4, 5, 6, 7, 8, 9, 10, 11, 12, 13, 14

#### Type locality.

Gwangot (33°33'10.37"N, 126°38'35.60"E), Jeju Island, Korea.

#### Type material.

***Holotype***. • One adult ♀ (MABIK CR00261201), preserved in a vial with 80% ethanol, collected from the type locality, 25 May 2024, by K Cho and JH Shin. ***Allotype***. • One adult ♂ (MABIK CR00261202), preserved in a vial with 80% ethanol, collection data as in holotype. ***Paratypes***. • Two adult ♀♀ (MABIK CR00261203, 261205) and one adult ♀ (MInRB-Hr108-S001), each dissected and mounted on three H-S slides; • two adult ♂♂ (MABIK CR00261204, 261206) and one adult ♂ (MInRB-Hr108-S003), each dissected and mounted on three H-S slides; • five adult ♀♀ and five adult ♂♂ on a stub for SEM; • two CV ♀♀ (MABIK CR00261207, 261209) and one CV ♀ (MInRB-Hr108-S007), each dissected and mounted on three H-S slides; • two CV ♂♂ (MABIK CR00261208, 261210) and one CV ♂ (MInRB-Hr108-S009), each dissected and mounted on three H-S slides; • five CV ♀♀ and three CV ♂♂ on a stub for SEM. Collection data as in holotype.

#### Other material examined.

• One adult ♀ (MInRB-Hr108-L001) and one adult ♂ (MInRB-Hr108-L002), preserved in a vial with 80% ethanol, collected near Deuksaenggot Lighthouse (33°31'29.41"N, 126°57'11.24"E), Udo Islet, Jeju Island, 31 July 2024, by J Lee, K Cho, and JH Shin; • one adult ♀ (MInRB-Hr108-L003) and one adult ♂ (MInRB-Hr108-L004), preserved in a vial with 80% ethanol, collected from Janghang port (34°59'38.00"N, 128°26'12.00"E), Goseong, 24 May 2020, by JG Kim; • one adult ♂ (MInRB-Hr108-L005), preserved in a vial with 80% ethanol, collected from Daenari port (37°00'22.15"N, 129°25'02.37"E), Uljin, 24 July 2024, by J Lee, K Cho, and JH Shin; • one adult ♀ (MInRB-Hr108-L006), preserved in a vial with 80% ethanol, collected from Mulgol (37°14'35.16"N, 131°51'51.37"E), West Islet, Dokdo Island, 27 May 2015, by HS Rho and JH Lee.

#### Differential diagnosis.

In the female, a distinctly seven-segmented antennule, a ventral cuticular expansion with an undulating edge on the first free abdominal somite, caudal rami with a C-shaped proximal dorsolateral expansion and auriform lateral elevation, and a markedly elongate inner seta on P4exp-2, ~ 2× as long as exp-2. In the male, a long inner seta on P2enp-2 and a hoe-shaped outer spine on P3enp-2.

#### Description.

**Figure 2.**
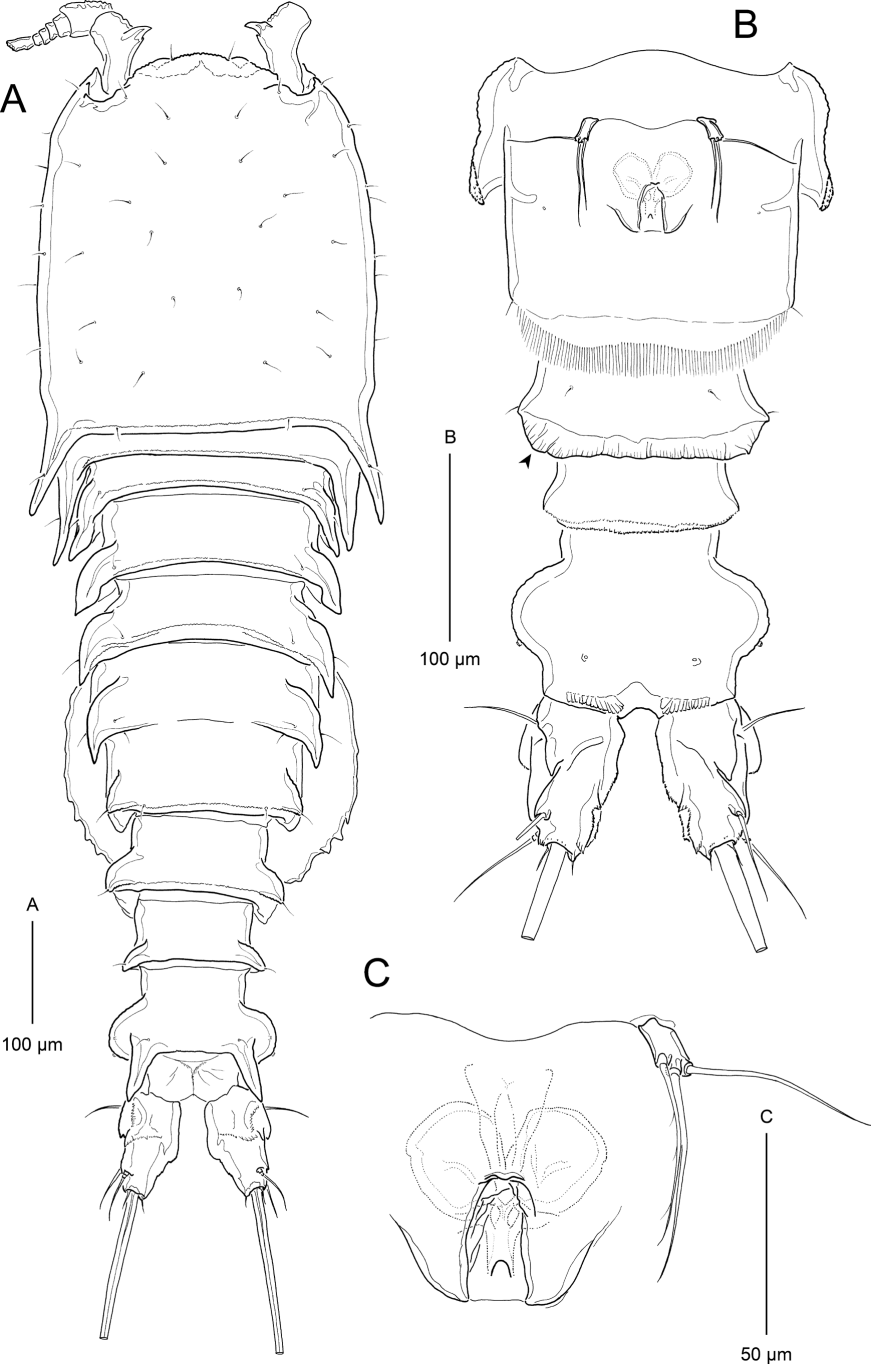
*Laophontella changi* sp. nov., holotype (MABIK CR00261201), adult female. **A**. Habitus, dorsal. Paratype (MABIK CR00261205), adult female: **B**. Urosome, ventral; **C**. Genital field.

**Adult female**. Body length (holotype, measured in lateral view from anterior margin of rostrum to posterior margin of caudal rami) 1.07 mm (range: 1.07–1.19 mm, *n* = 12). Habitus (Figs 2A, 12A) subcylindrical, gradually tapering posteriorly; integument strongly chitinized. Prosome (Fig. 2A) slightly shorter than urosome, comprising the cephalothorax (cephalosome completely fused with first pedigerous somite) and three free pedigerous somites. Each somite bearing a pair of pointed posterolateral processes, decreasing in size towards posterior somite; posterior margin finely serrated due to surface ornamentation. Cephalothorax (Figs 2A, 12B) rectangular, slightly longer than wide, occupying ~ 34% of body length (measured excluding posterolateral process); dorsal surface covered with numerous, well-defined cuticular pores of various sizes, together with integumental pores and sensilla as figured. Surface of free prosomites with numerous cuticular pits and scattered denticles. P2-bearing somite with pointed posterolateral processes as long as those on cephalothorax. P3- and P4-bearing somites with relatively short posterolateral processes.

#### Urosome

(Figs 2A, 2B, 12C) comprising P5-bearing somite, genital double-somite, and three free abdominal somites; dorsal surface ornamented with several sensilla, minute pits, and scattered denticles. Dorsal posterior margins of somites finely serrated due to surface ornamentation. Genital somite and first abdominal somite fused, forming genital double-somite; original segmentation marked by distinct dorsolateral surface ridge and two pairs of small lateral projections, the latter causing the lateral margins to appear partially separated; ventral surface with a pair of pores; ventral distal margin with a fringe of long setules (Fig. 2B). Genital field (Fig. 2C) located medially on anterior half of genital double-somite, with a copulatory pore, and the area surrounding the pore slightly protruding on both sides. P6 (Fig. 2C) represented by a small segment bearing one outer naked and two apical setae, the inner one unipinnate. First free abdominal somite (Figs 2A, 2B, 12C) with distal margin distinctly flared and slightly expanded laterally; with a ventral cuticular expansion bearing an undulating edge (indicated by arrowhead in Figs 2B, 12C). Second free abdominal somite (Figs 2A, 2B, 12C) smaller than the first dorsally; ventral margin finely serrated due to surface ornamentation. Anal somite (Figs 2A, 2B, 7A, 7B, 12C) laterally expanded, with prominently rounded lateral margins, shorter than maximum width; posteroventral margin serrated due to surface ornamentation (Figs 7A, 13B); dorsal surface with one pair of sensilla, two pairs of lateral pores, one pair of ventral pores, and prominent posterolateral processes. Anal operculum ornamented with fine spinules.

***Caudal rami*** (Figs 2A, 2B, 7A, 7B, 13A–C) ~ 1.6× as long as maximum width; proximal dorsolateral region distinctly expanded (C-shaped in lateral view, indicated by ‘a’ in Figs 7A, 7B, 13A, 13C); lateral margin with a broad auriform lateral elevation (indicated by ‘b’ in Figs 7A, 7B, 13A–C); with a pronounced ventrolateral bulge extending from proximal region toward insertion of seta III, bulge interrupted medially to form a short acute projection; inner margin distinctly concave. Surface covered with numerous denticles and scattered pits; with three pores, one each on dorsal, ventral, and lateral surfaces; with seven setae: ventrolateral setae I and II inserted in proximal third of the ramus, the former strongly reduced, the latter 4× the length of seta I; seta III near the distal outer corner, slightly longer than seta II; terminal setae IV and VI short, reduced; seta IV basally fused with seta V; seta V well-developed, longest, ~ 5× as long as seta II; seta VII tri-articulate, arising dorsally at distal ~ 1/6 of the ramus length.

***Rostrum*** (Fig. 2A) broad, completely fused to the cephalothorax, with a pair of apicolateral sensilla. Integumental ornamentation similar to that of the dorsal surface of cephalothorax.

**Figure 3.**
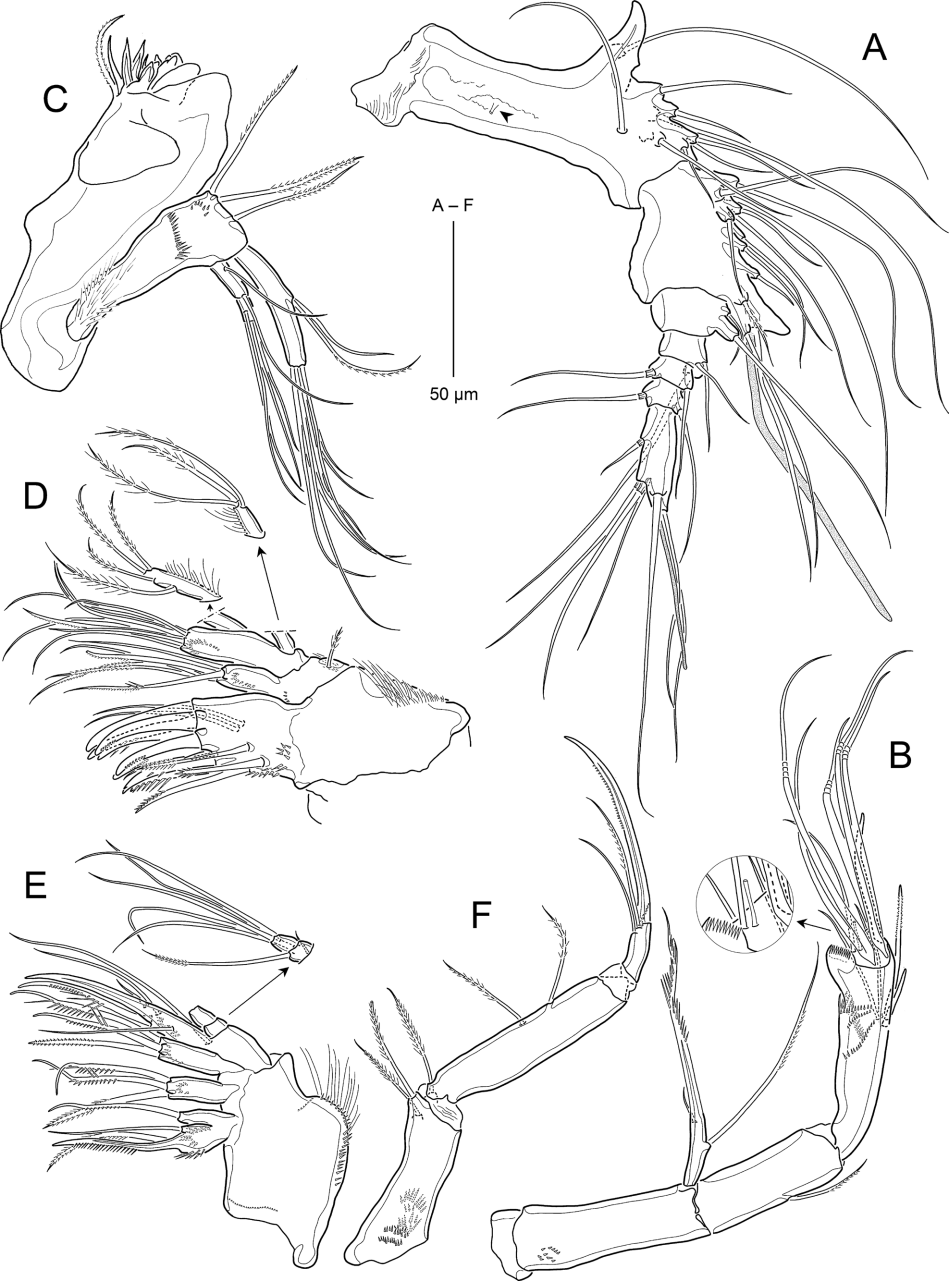
*Laophontella changi* sp. nov., paratype (MABIK CR00261205), adult female. **A**. Antennule; **B**. Antenna; **C**. Mandible; **D**. Maxillule; **E**. Maxilla; **F**. Maxilliped.

***Antennule*** (Fig. 3A) seven-segmented. First segment longest, with a bent process at distal ~ 1/3 of inner margin and a tube pore (indicated by arrowhead in Fig. 3A). Second segment half as long as first segment, bearing an aesthetasc fused to a naked seta on the distal inner process. Fifth segment shortest. Seventh segment distally with a small aesthetasc fused to two naked setae. Armature formula: 1-[10], 2-[11 + (1 + ae)], 3-[2], 4-[3], 5-[2], 6-[2], 7-[5 + acrothek].

***Antenna*** (Fig. 3B). Coxa small and unornamented. Basis elongate, ornamented with several minute inner spinules. Endopod two-segmented; proximal segment slightly shorter than basis, anteriorly partially fused to basis, bearing one abexopodal seta at mid-inner margin; distal segment slightly longer than basis, ornamented with several rows of minute spinules, two outer subdistal hyaline frills, and one distal tube pore; with following armature: laterally two setae and two spines; distally one spine, four geniculate, and two naked setae, the two outermost distal setae basally fused. Exopod arising distally on basis, one-segmented, with one lateral and two distal setae; outermost distal seta basally fused to segment and distally spinulose.

***Mandible*** (Fig. 3C). Coxa long, bearing a fin-like process; gnathobase well-developed, with two blunt teeth, several uni- and multi-cuspidate teeth, and one pinnate seta. Palp biramous, consisting of basis, two-segmented exopod, and one-segmented endopod. Basis elongated, gradually widening distally, with three pinnate setae, and ornamented with two rows of spinules and several setules on anterior surface. Exopod shorter than endopod; proximal segment with two naked setae; distal segment smaller than proximal segment, with four naked setae (one subdistal and three terminal). Endopod one-segmented, with two lateral (one pinnate and one naked) and seven terminal setae.

***Maxillule*** (Fig. 3D). Praecoxa ornamented with outer setules; arthrite well-developed, with two juxtaposed anterior naked setae, eight distal spines, one distal naked, one subdistal pinnate, and two posterior pinnate setae; posterior surface with several spinules. Coxa with anterior outer setules and posterior inner spinules; coxal epipodite represented by one bipinnate seta; coxal endite cylindrical, with two naked and four pinnate setae, and a row of anterior spinules. Basis with a row of anterior spinules, and with eight setae. Exopod one-segmented, with inner setules and three distal plumose setae. Endopod one-segmented, with outer setules and four setae.

***Maxilla*** (Fig. 3E). Syncoxa large, ornamented with a row of spinules and a row of setules along outer margin, and two rows of denticles on anterior surface; with three endites: proximal endite bilobed, proximal lobe with one naked and two pinnate setae, the anterior seta basally fused to lobe, distal lobe with one pinnate seta; middle and distal endites each with three pinnate setae; each endite ornamented posteriorly with a row of spinules. Allobasis drawn out into a curved claw, with one spinulose and three naked setae distally (one short and two long), and one long naked seta proximally. Endopod two-segmented; proximal segment with one pinnate and two naked setae, one of these latter very short; distal segment with four naked setae.

***Maxilliped*** (Fig. 3F) subchelate, comprising syncoxa, basis, and one-segmented endopod. Syncoxa elongate, ornamented anteriorly and posteriorly with rows of various-sized spinules, and with three bipinnate setae on distal inner corner. Basis longer than syncoxa, with two bipinnate setae at mid-inner margin. Endopod with a claw bearing a row of minute spinules along distal half of inner concave margin, and with one unipinnate and two naked setae, one of these latter very short.

**Figure 4.**
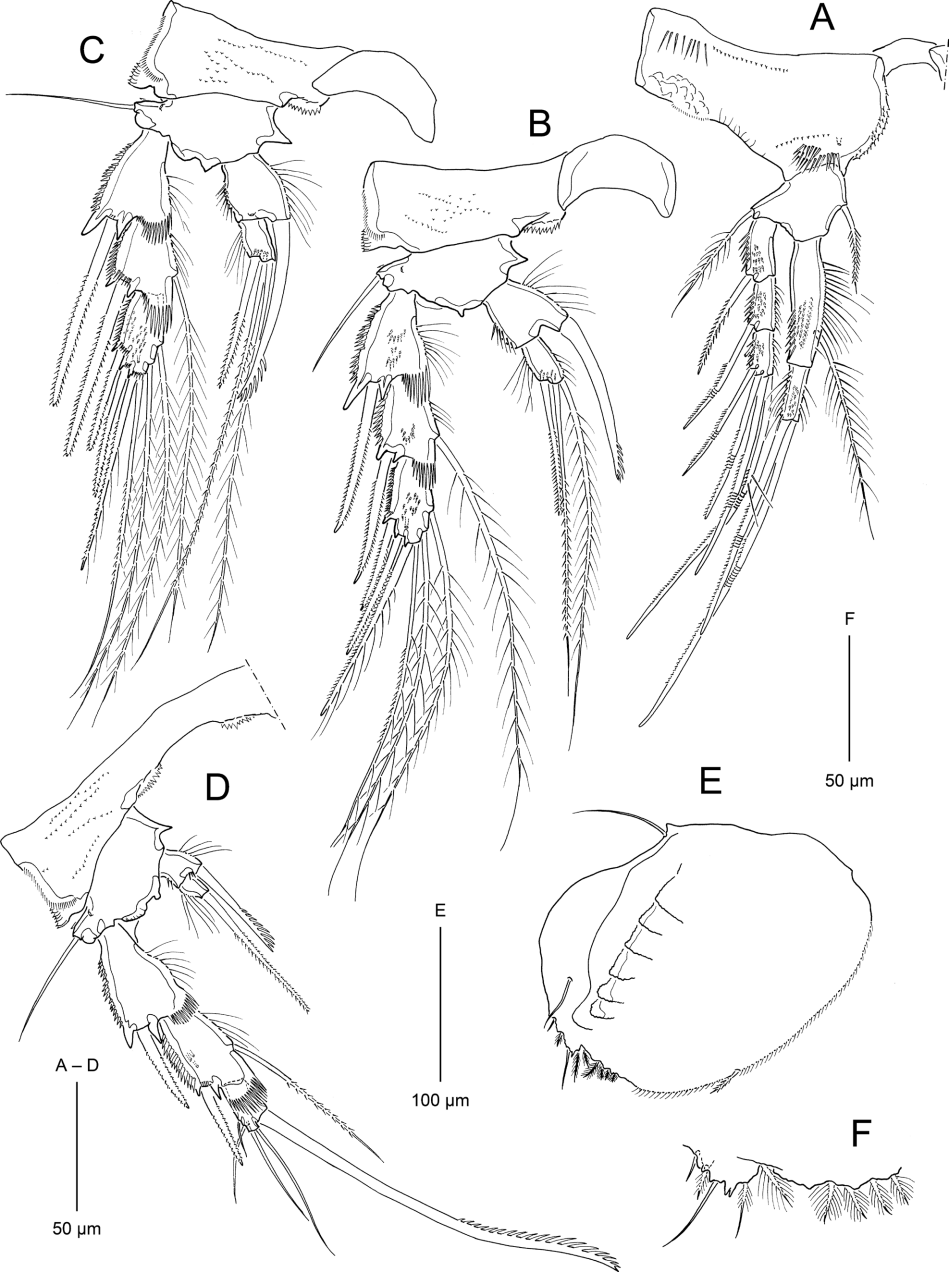
*Laophontella changi* sp. nov., paratype (MABIK CR00261205), adult female. **A**. P1, anterior; **B**. P2, anterior; **C**. P3, anterior; **D**. P4, anterior; **E**. P5, anterior; **F**. Distal margin of P5.

***P1*** (Fig. 4A). Intercoxal sclerite small, subrectangular. Coxa large, laterally elongate; anterior surface with reticulate sculpture and several denticles; a proximal row of setules near outer margin, several distal rows of spinules and a pore near the basis. Basis much smaller than coxa, articulated to inner margin of coxa, with one inner bipinnate and one outer plumose seta. Exopod three-segmented, each segment with outer marginal and posterior spinules; exp-1 without inner setules and with one outer bipinnate seta; exp-2 with inner setules and one outer geniculate unipinnate seta; exp-3 with inner setules, one outer tube pore, and four geniculate unipinnate setae (two outer and two distal). Endopod two-segmented, both segments with posterior spinules; enp-1 with inner setules, outer spinules, and one inner plumose seta; enp-2 with inner and outer setules, and two distal geniculate unipinnate setae.

***P2*** (Fig. 4B). Intercoxal sclerite subrectangular. Coxa rectangular, ornamented with a reticulate pattern on anterior surface, with spinules along outer margin. Basis smaller than coxa and laterally wider than that of P1; anterior surface with an acute inner projection, and with one pore near outer basal naked seta; distal margin with a minute acute projection between rami. Exopod three-segmented, each segment with outer marginal and posterior spinules; exp-1 with inner setules, a large distal hyaline frill, and one outer bipinnate spine; exp-2 with inner setules, a large distal hyaline frill, one outer bipinnate spine, and one inner plumose seta; exp-3 with one anterodistal pore, two outer bipinnate and one apical bipinnate spine, one apical bipinnate, and two inner plumose setae. Endopod two-segmented; enp-1 with inner setules and outer spinules, bearing one thick inner seta, unilaterally pectinate distally; enp-2 with inner and outer setules, and posterior spinules, bearing two bipinnate (one outer and one apical) and one apical plumose seta.

***P3*** (Fig. 4C). Intercoxal sclerite trapezoidal. Coxa as in P2. Basis as in P2, except with two larger projections on the distal margin. Exopod as in P2, except exp-1 and exp-2 lacking spinules on posterior surface, and each with outer spine longer than in P2. Endopod as in P2, except enp-1 with an anterodistal pore.

***P4*** (Fig. 4D). Intercoxal sclerite fused to the coxa. Coxa asymmetrically trapezoidal, inner margin narrow and outer margin distinctly broader; ornamentation as in P2. Basis as in P2. Exopod three-segmented; exp-1 and exp-2 as in P3, but with shorter outer spines; exp-2 with three anterior pores; exp-3 very short, with one anterodistal pore, four slender naked (two outer and two apical), and one very long thick seta, the distal half of the latter unilaterally pectinate. Endopod two-segmented, reduced compared to those of P2 and P3; enp-1 with inner setules, and distal and outer spinules, bearing one inner seta, the distal half of the latter unilaterally pectinate; enp-2 with outer setules, bearing one apical bipinnate seta. Armature formulae of P1–P4 as follows:

**Table:** 

**Leg**	**Exopod**	**Endopod**
P1	0.0.022	1.020
P2	0.1.222	1.021
P3	0.1.222	1.021
P4	0.1.122	1.010

***P5*** (Figs 4E, 12A) foliaceous, with baseoendopod and exopod completely fused, a bulge on outer edge, and one outer proximal seta (outer basal seta); inner edge with a fringe of short setules. Surface ornamentation regionally differentiated (Fig. 12A): inner area partly smooth and distally denticulate; median and outer surfaces densely denticulate with lamellate, terraced folds; outer margin with a reticulate pattern. Distally armed with 11 setae: six setae of exopodal origin, comprising one outer subdistal naked, and five setae along distal margin, and five setae of endopodal origin, including one short bipinnate seta at ~ 2/3 of inner margin; distal margin (Fig. 4F) with a minute bifid process between third and fourth setae of the distal series.

**Figure 5.**
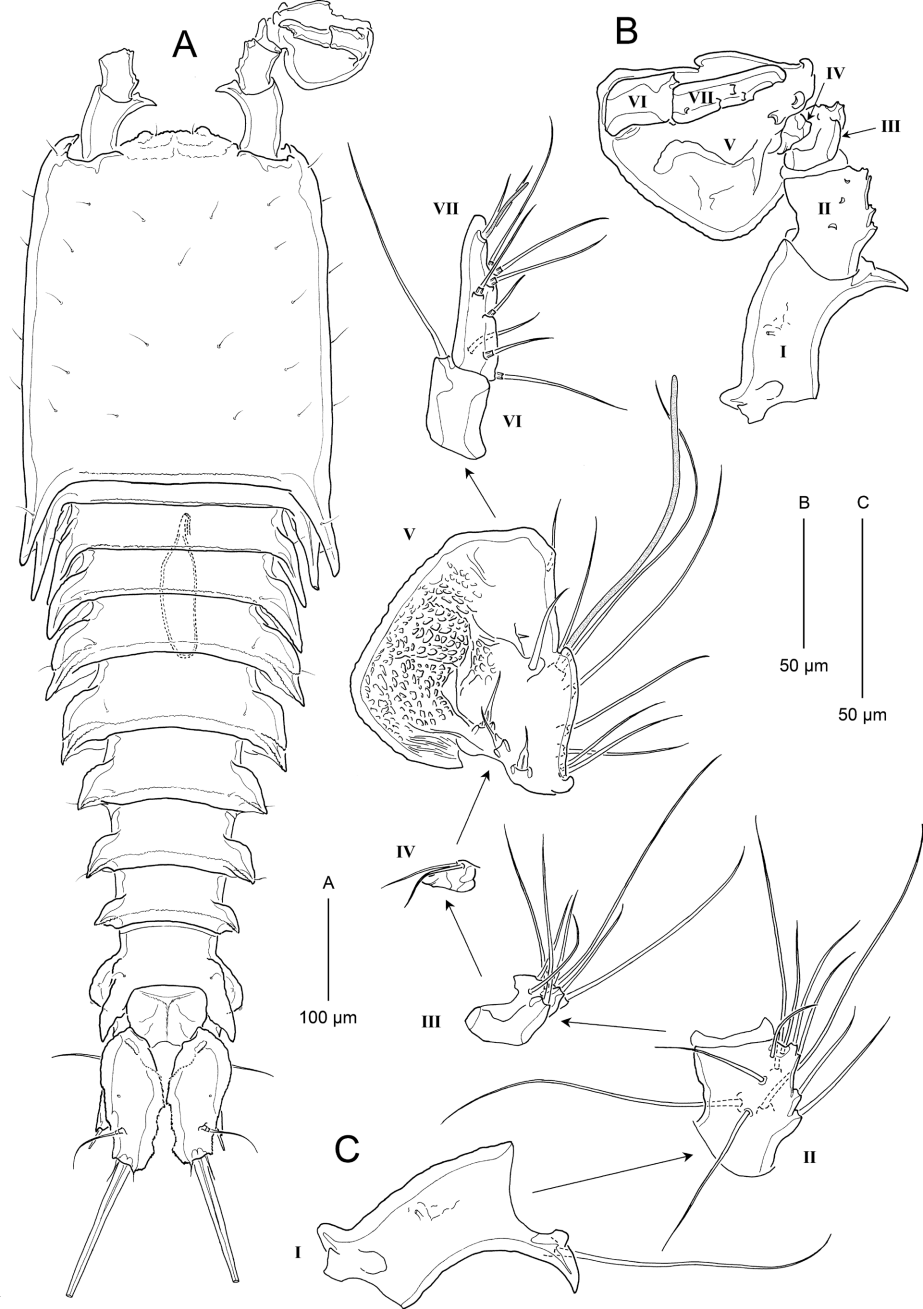
*Laophontella changi* sp. nov., allotype (MABIK CR00261202), adult male. **A**. Habitus, dorsal. Paratype (MABIK CR00261206), adult male: **B**. Antennular segmentation; **C**. Antennule.

**Adult male**. Body length (allotype, measured in lateral view) 0.83 mm (range: 0.83–0.97 mm, *n* = 12). Habitus (Figs 5A, 12D) as in the female except for urosomal segmentation with second and third urosomites separated. Sexual dimorphism also observed in caudal rami, antennule, and P2–P6.

**Figure 6.**
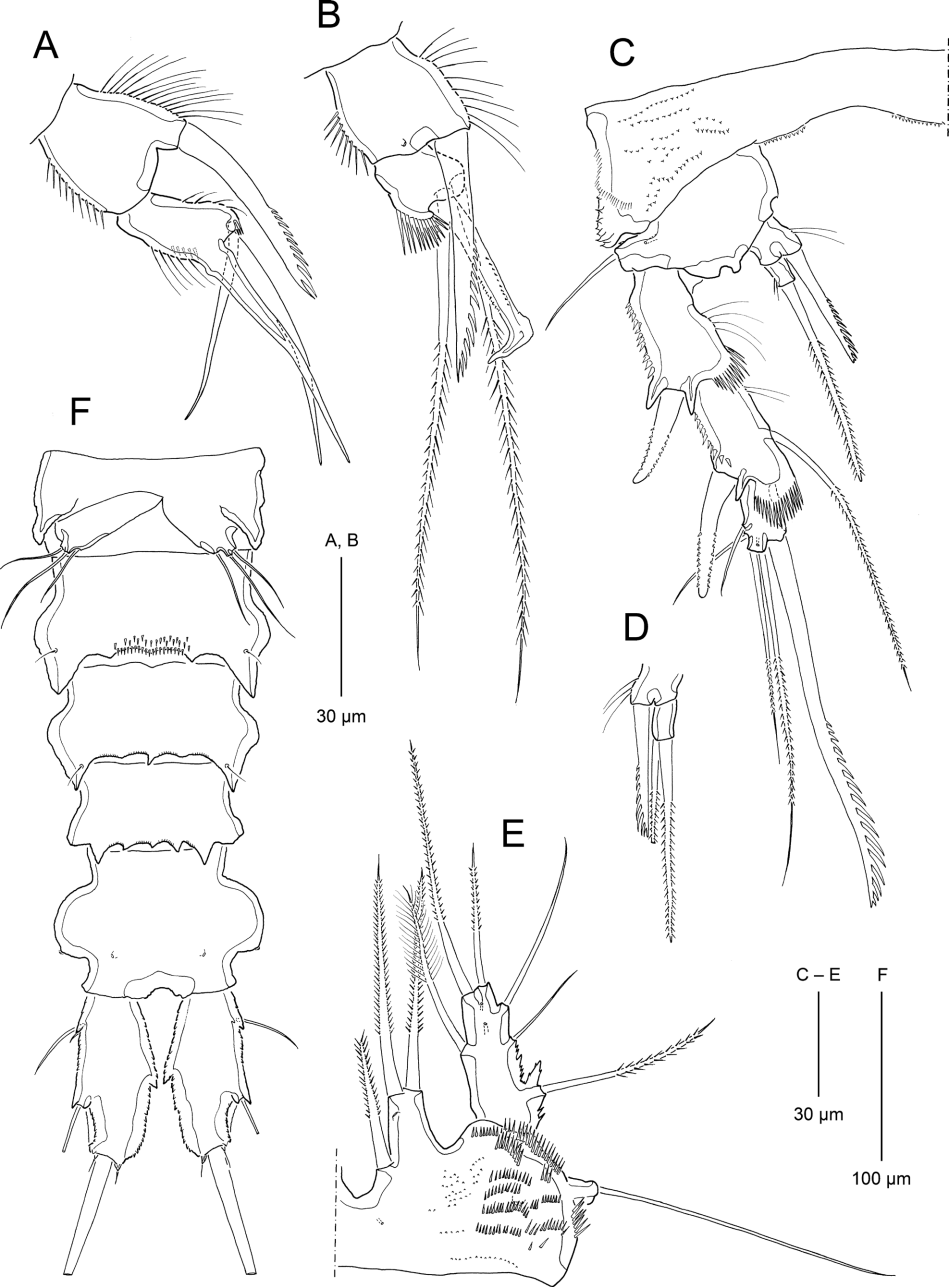
*Laophontella changi* sp. nov., paratype (MABIK CR00261206), adult male. **A**. P2 endopod, anterior; **B**. P3 endopod, anterior; **C**. P4, anterior; **D**. Variation of P4 endopod, anterior; **E**. P5, anterior; **F**. Urosome, ventral.

***Urosome*** (Figs 5A, 6F, 12E) six-segmented with ornamentation as figured. First free abdominal somite with a patch of spinules along posteroventral margin (Fig. 6F). First to third free abdominal somites with weakly crenulate projections along posteroventral margins (Figs 6F, 12E).

**Figure 7.**
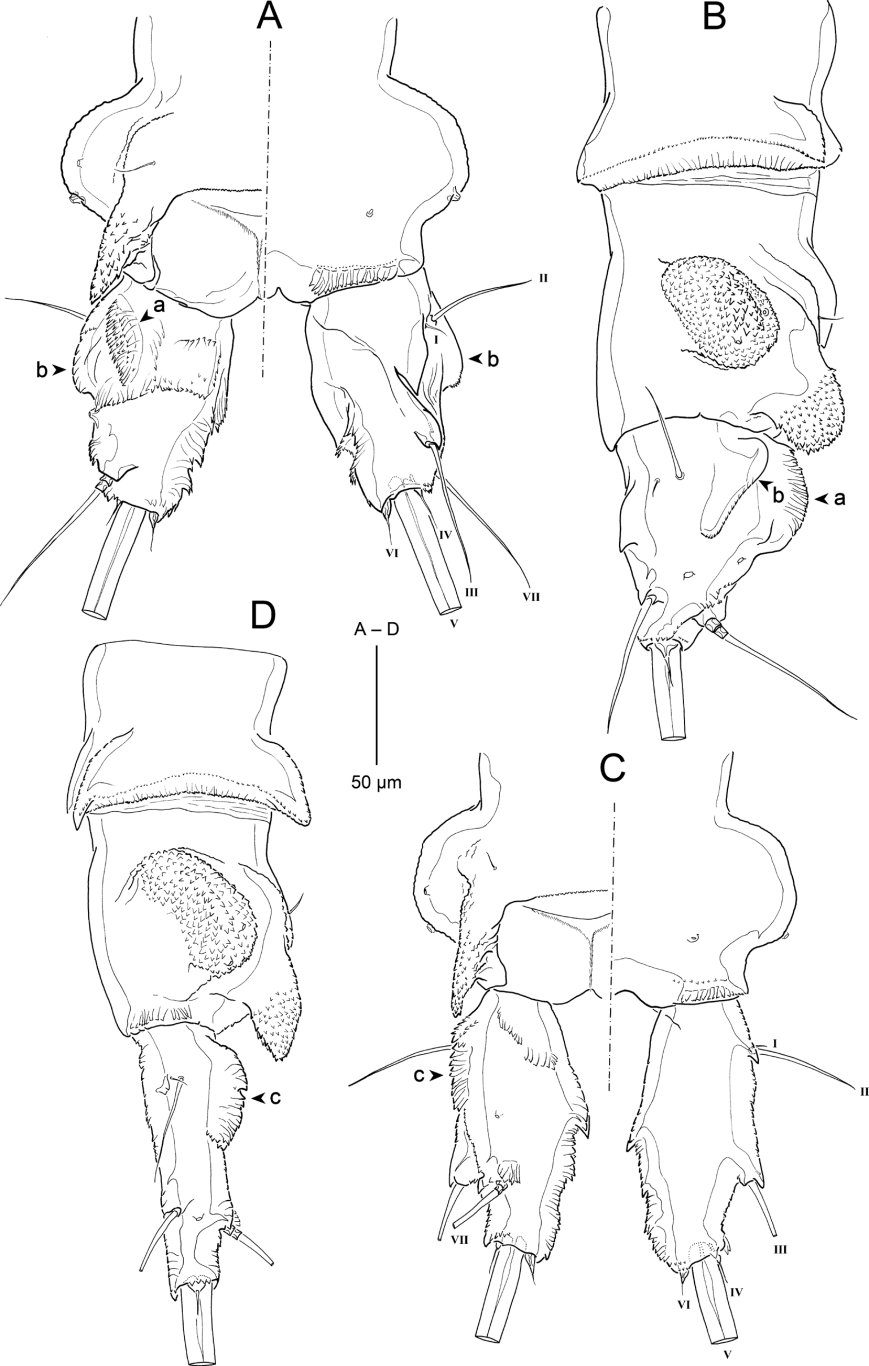
*Laophontella changi* sp. nov., paratype (MABIK CR00261205), adult female. **A**. Anal somite and caudal ramus, dorsal (left) and ventral (right); **B**. Second free abdominal somite, anal somite, and caudal ramus, lateral. Paratype (MABIK CR00261206), adult male: **C**. Anal somite and caudal ramus, dorsal (left) and ventral (right); **D**. Third free abdominal somite, anal somite, and caudal ramus, lateral. **a, c**. A dorsolateral expansion; **b**. A lateral process.

***Caudal rami*** (Figs 5A, 6F, 7C, 7D, 13D–F) ~ 2× as long as wide, slenderer and more cylindrical than in the female, proximal outer margin with weak cuticular expansions dorsolaterally (indicated by ‘c’ in Figs 7C, 7D, 13D–F); surface ornamentation similar to that of the female; caudal seta III inserted more laterally than in the female; seta VII arising dorsally at distal ~ 1/4 of ramus length.

***Antennule*** (Fig. 5B, C) seven-segmented, subchirocer, with geniculation between fifth and sixth segments. First segment with a bent process and a tube pore as in the female. Fourth segment smallest. Fifth segment distinctly swollen, inner surface with a concavity; inner margin with two small triangular projections; surface irregularly reticulate. Seventh segment cylindroconical, with a small aesthetasc basally fused to two naked setae. Armature formula: 1-[1], 2-[11], 3-[8], 4-[2], 5-[11 + (1 + ae)], 6-[1], 7-[8 + (acrothek)].

***P2*** exopod as in the female. Enp-1 (Fig. 6A) with inner seta slightly thicker than that of the female. Enp-2 (Fig. 6A) with two naked and robust apical setae, both shorter than those of the female, and the outer one basally fused to the segment. Inner seta naked and shorter than that of the female.

***P3*** exopod as in the female. Enp-1 (Fig. 6B) with inner seta slightly thicker than that of the female. Enp-2 (Fig. 6B) with an outer marginal spinular row; two apical setae similar to those of the female; and one modified outer spine, medially bipinnate and hoe-shaped.

***P4*** (Fig. 6C). Basis inner projection minute and reduced. Exopod and endopod as in the female, except for the robust outer spines on exp-1 and exp-2, the longer two apical bipinnate setae on exp-3, and the presence of outer marginal spinules on enp-2.

***P5*** (Fig. 6E) not foliaceous, smaller than that of the female. Baseoendopods of right and left legs fused medially; anterior surface with one pore and several rows of spinules and denticles, bearing one outer basal seta. Endopodal lobe distinctly protruded, cylindrical, reaching ~ 1/2 the length of exopod, with one anterodistal pore and three bipinnate setae (one inner and two distal). Exopod one-segmented, ~ 2× as long as maximum width; with two anterodistal pores; proximal half of outer margin strongly dentate and bearing an acute outer process below outer proximal seta; armature consisting of one outer bipinnate, two outer naked, two apical bipinnate, and one inner plumose seta.

***P6*** (Fig. 6F) asymmetrical, with one plate fused to ventral wall of supporting somite and the opposite plate free; distal outer corner with three naked setae.

**Figure 8.**
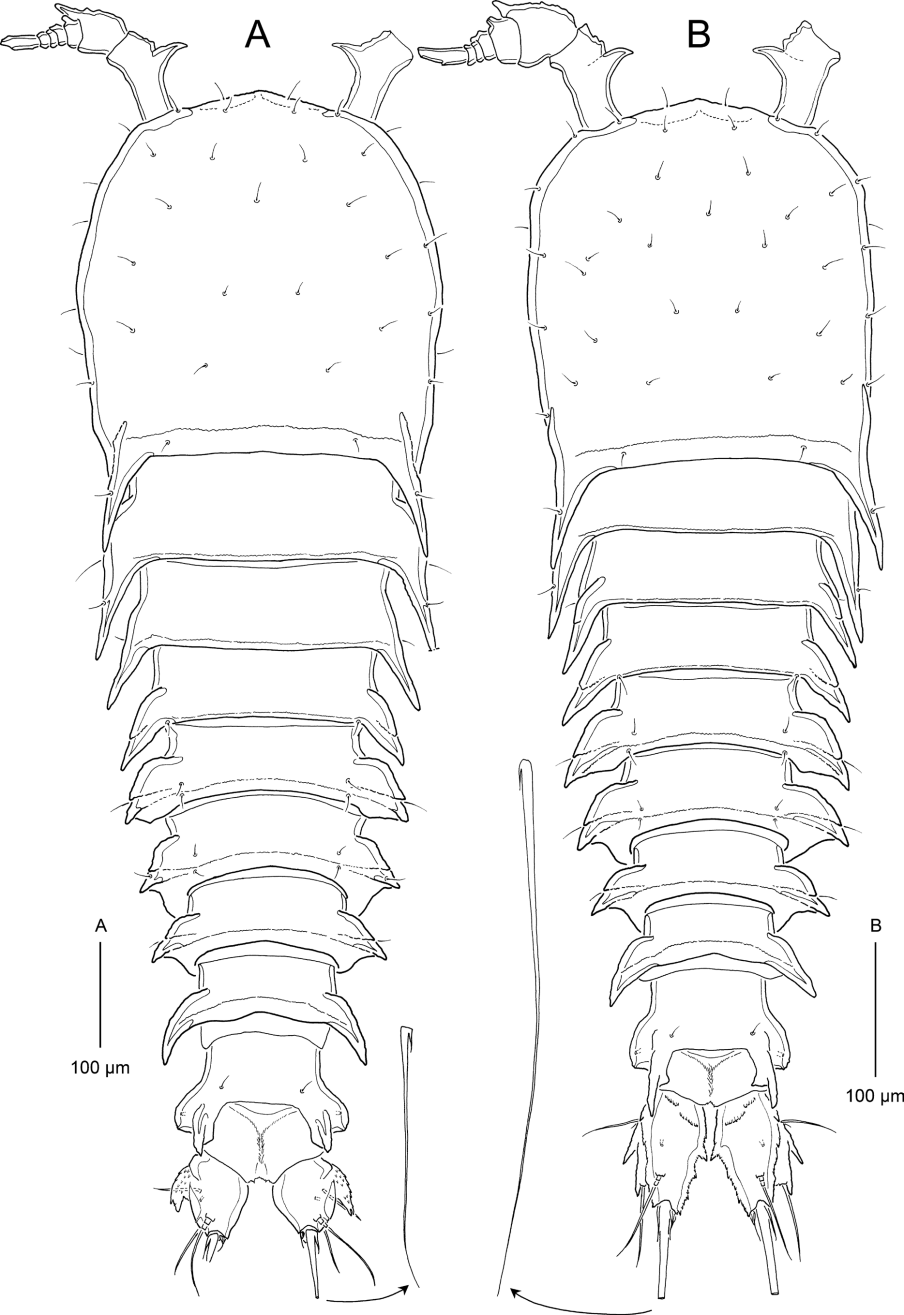
*Laophontella changi* sp. nov., paratype (MABIK CR00261209), copepodid V female. **A**. Habitus, dorsal. Paratype (MABIK CR00261210), copepodid V male: **B**. Habitus, dorsal.

**CV Female**. Body length (paratype, measured in lateral view from anterior margin of rostrum to posterior margin of caudal rami) 0.95 mm (range: 0.85–0.95 mm, *n* = 8). Habitus (Fig. 8A) generally as in the adult female, subcylindrical, gradually tapering posteriorly, but body composed of nine somites. Prosome (Fig. 8A) with each somite bearing a pair of pointed posterolateral processes; these processes proportionally longer and more strongly developed than in the adult female. Dorsal surface of the cephalothorax covered with fewer and less well-developed cuticular pores than in the adult female.

***Urosome*** (Figs 8A, 10E, 12F) comprising P5-bearing somite, genital somite, and three free abdominal somites; dorsal surface ornamentation and posterior marginal shape of urosomites as in the adult female. Urosomites dorsally with distal margins distinctly flared and laterally expanded, more strongly than those of the adult female. P6 (Fig. 10E) represented by a small triangular plate, fused to somite, bearing three setae. First and second free abdominal somites (Figs 10E, 12F) with weakly crenulate projections along posteroventral margins. Anal somite (Figs 8A, 10E, 11A, 11B, 12F) laterally less expanded than that of the adult female; lateral margins obliquely rounded; other features as in the adult female.

***Caudal rami*** (Figs 8A, 10E, 11A, 11B, 14A–C) ~ 1.1× as long as maximum width; proximal dorsolateral region (indicated by ‘a’ in Figs 11A, 11B, 14A, 14C) expanded and weakly ornamented; outer margin in proximal half laterally produced into a wing-shaped projection (indicated by ‘b’ in Figs 11A, 11B, 14A–C), bearing several denticles; with pronounced ventrolateral bulge from proximal region toward insertion of seta III. Surface mostly smooth except for a dorsolateral expansion and a wing-shaped projection; with three pores, each one on dorsal, ventral, and lateral surfaces; with seven setae: ventrolateral setae I and II inserted in proximal third of the ramus; seta III near the distal outer corner on ventral side; setae IV and VI short, reduced; seta IV basally fused with seta V; seta V well-developed, longest; seta VII tri-articulate, arising dorsally at distal ~ 1/5 of the ramus length.

Antennule, antenna, and mouthparts as in the adult.

**Figure 9.**
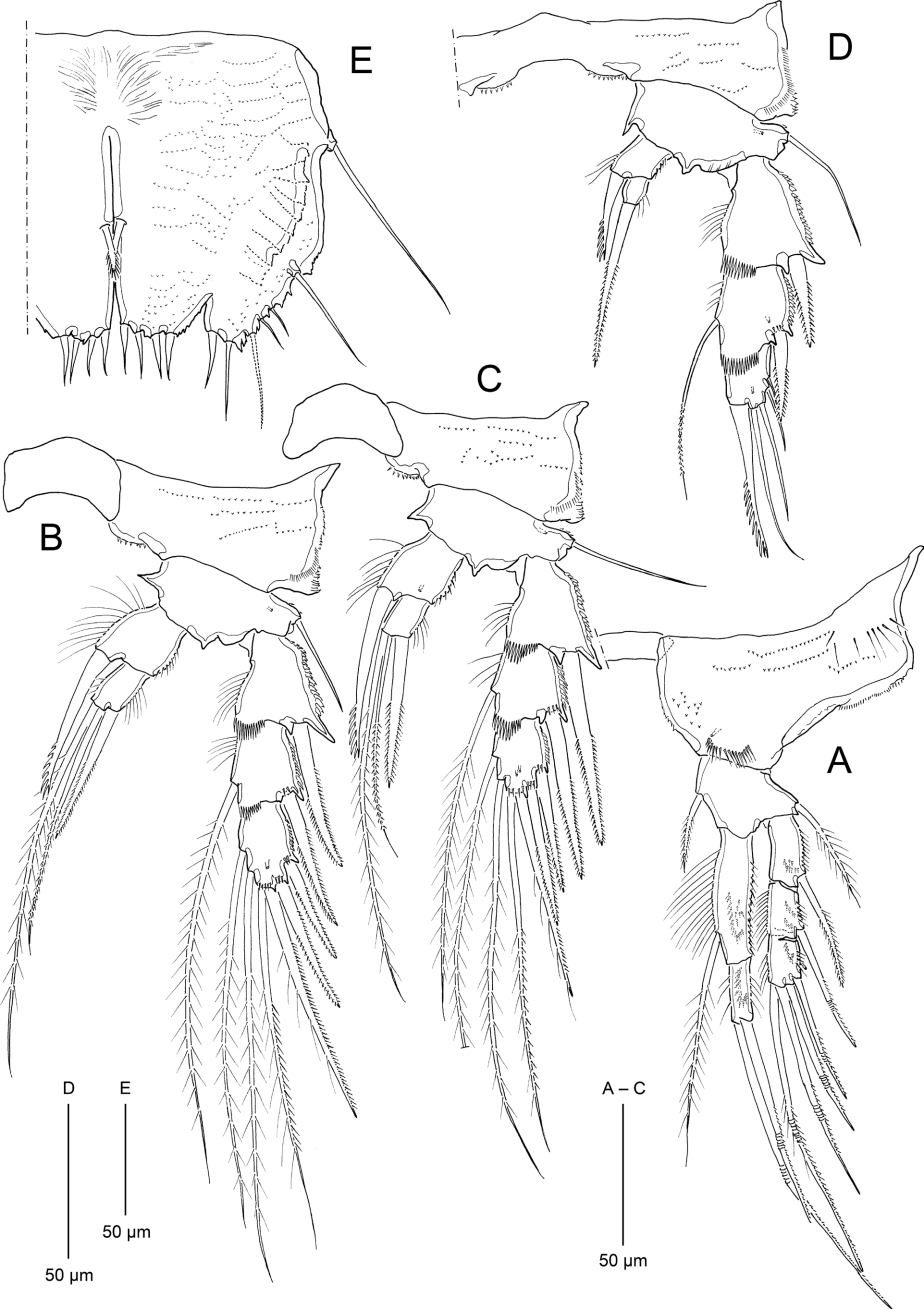
*Laophontella changi* sp. nov., paratype (MABIK CR00261209), copepodid V female. **A**. P1, anterior; **B**. P2, anterior; **C**. P3, anterior; **D**. P4, anterior; **E**. P5, anterior.

***P1–P3*** (Fig. 9A–C) as in the adult female, except that exopodal segments in P2 and P3 relatively broader than those of the adult, and the outer apical seta of enp-2 relatively shorter than in the adult; segmentation of P1 exp-2–3 variable (see Variability).

**Figure 10.**
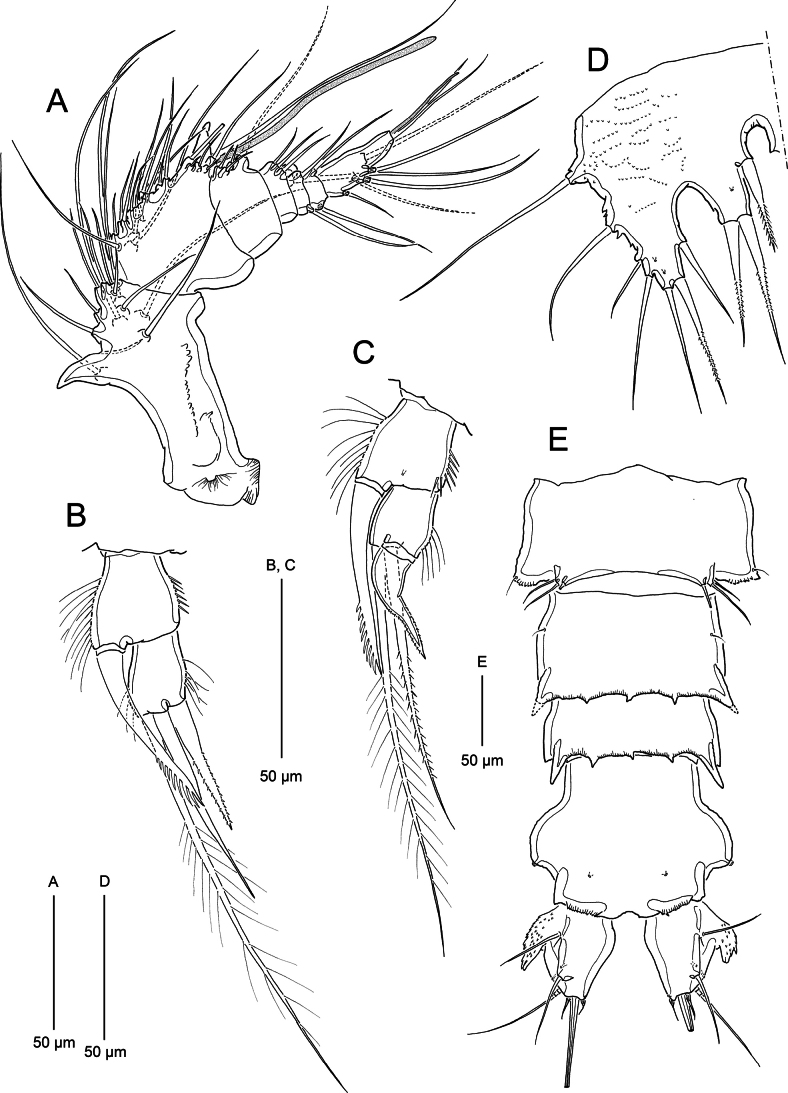
*Laophontella changi* sp. nov., paratype (MABIK CR00261210), copepodid V male. **A**. Antennule; **B**. P2 endopod, anterior; **C**. P3 endopod, anterior; **D**. P5, anterior. Paratype (MABIK CR00261209), copepodid V female: **E**. Urosome, ventral.

**Figure 11.**
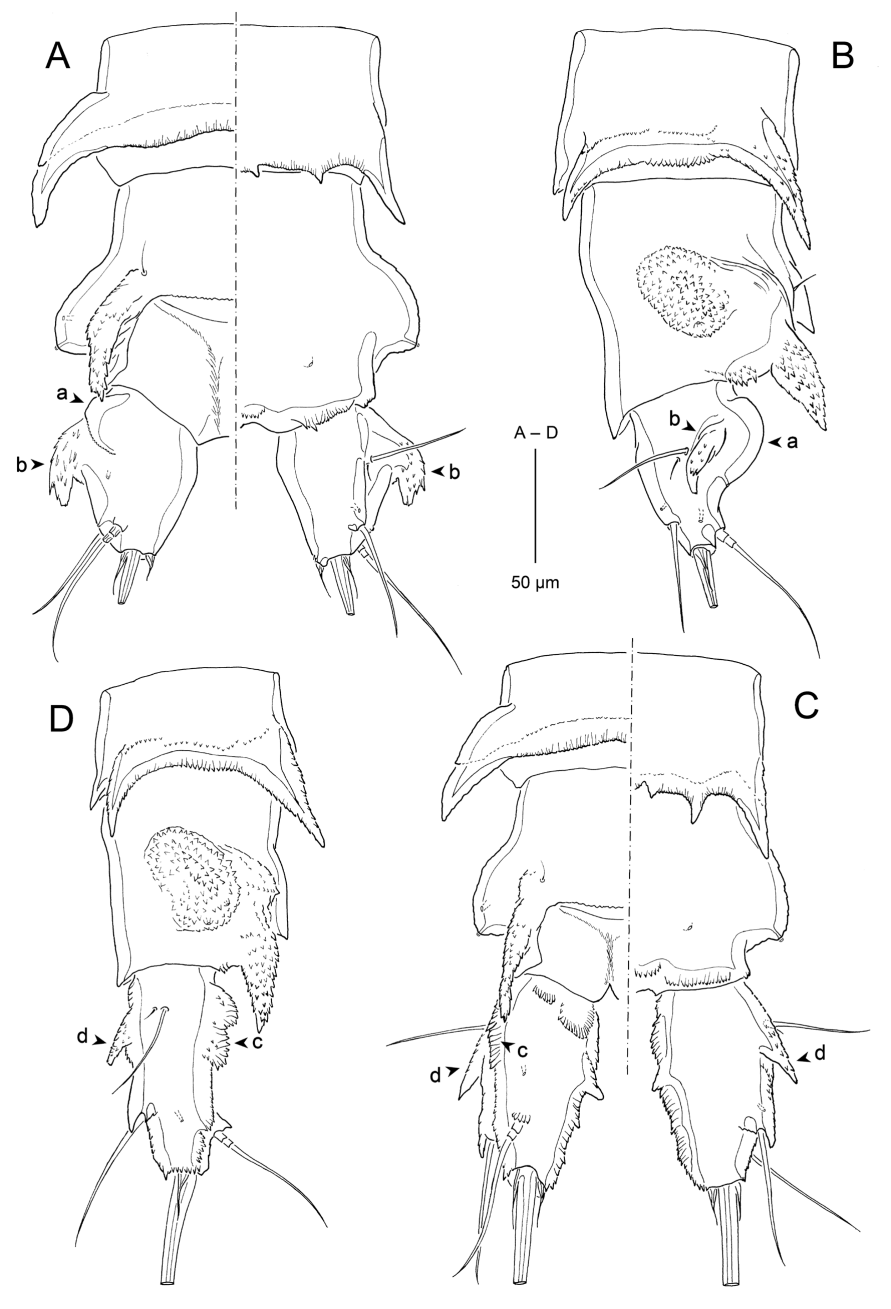
*Laophontella changi* sp. nov., paratype (MABIK CR00261209), copepodid V female. **A**. Second free abdominal somite, anal somite, and caudal ramus, dorsal (left) and ventral (right); **B**. Same, lateral. Paratype (MABIK CR00261210), copepodid V male: **C**. Third free abdominal somite, anal somite, and caudal ramus, dorsal (left) and ventral (right); **D**. Same, lateral. **a, c**. A dorsolateral expansion; **b**. A lateral process; **d**. A ventrolateral process.

**Figure 12.**
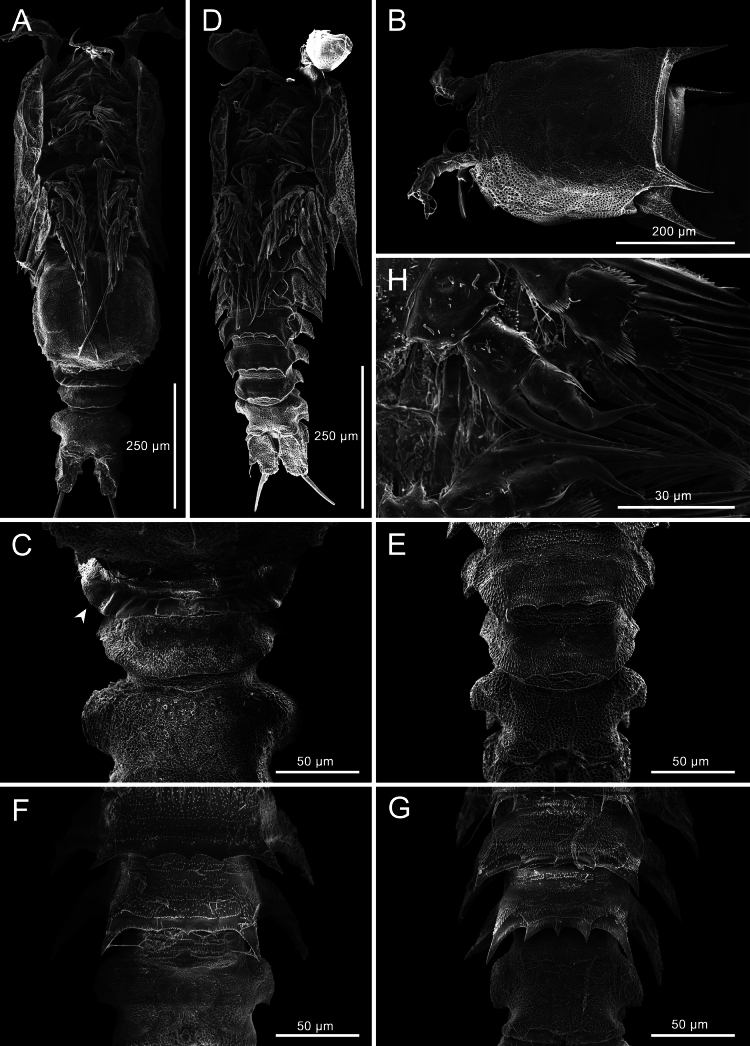
Scanning electron micrographs of *Laophontella changi* sp. nov., adult female. **A**. Habitus, ventral; **B**. Cephalothorax, dorsal; **C**. Three free abdominal somites, ventral. Adult male: **D**. Habitus, ventral; **E**. Three free abdominal somites, ventral. Copepodid V female: **F**. Three free abdominal somites, ventral. Copepodid V male: **G**. Three free abdominal somites, ventral; **H**. P3 endopod, anterior.

***P4*** (Fig. 9D) as in the adult female, except for exopodal segments broader than those of the adult, and the thick inner seta on exp-3 much shorter than that of the adult.

***P5*** (Fig. 9E) represented by weakly foliaceous plates. Baseoendopods of right and left legs fused medially. Baseoendopod and exopod incorporated into a single plate; baseoendopodal and exopodal portions distinguishable by a distal cleft (notch). Surface ornamentation generally smooth and partly denticulate; outer surfaces denticulate, with lamellate, terraced folds. Armed with 12 setae insertions as in the adult female; all setae naked except for one short bipinnate seta located at ~ 2/3 of inner margin.

**CV Male**. Body length (paratype, measured in dorsal view) 0.82 mm (range: 0.82–0.84 mm, *n* = 6). Habitus (Fig. 8B) as in the CV female. Sexual dimorphism detected in urosome, caudal rami, antennule, and P2–P5.

***Urosome*** (Figs 8B, 12G) generally as in the CV female, five-segmented. First and second free abdominal somites (Fig. 12G) with posteroventral margins more strongly crenulate than those of the CV female.

***Caudal rami*** (Figs 8B, 11C, 11D, 14D–F) ~ 1.5× as long as wide; overall shape and surface ornamentation generally as in the adult male, except for a less pronounced dorsolateral expansion (indicated by ‘c’ in Figs 11C, 11D, 14D, 14E) and an acute, denticulate projection (indicated by ‘d’ in Figs 11C, 11D, 14D–F) on outer margin in its proximal half.

***Antennule*** (Fig. 10A) seven-segmented, similar to that of the CV female, except for the second and third segments swollen, the first segment with a more prominent posterior suture line, and differences in armature of the first to fourth segments. Armature formula: 1-[12], 2-[19 + (1 + ae)], 3-[4], 4-[4], 5-[2], 6-[2], 7-[5 + acrothek].

***P2*** exopod as in the CV female. Enp-2 (Fig. 10B) broader than in the CV female; outer apical seta shorter and thickened.

***P3*** exopod as in the CV female. Enp-2 (Figs 10C, 12H) broader than in the CV female; outer spine modified, broadened basally, slightly bent outward, abruptly tapering from mid-length, distal half bipinnate.

***P5*** (Fig. 10D) generally as in the adult male in overall shape, but baseoendopod and exopod still fused; exopod not separated as a distinct lobe as in the adult male, and a process at base of proximal outer seta smaller than in the adult. Baseoendopod surface denticulate.

#### Variability.

Morphological variation in *L. changi* sp. nov. was observed in the armature of P4 in the adult male and in the segmentation/armature of P1 in the copepodid V (CV). In the typical condition, P4enp-2 bears a single terminal seta, but the left endopod of P4 has two terminal setae in one adult male specimen (Fig. 6D). All other examined adult males (*n* = 6) show the typical condition. In the CV stage (females *n* = 3, males *n* = 3), the articulation between exp-2 and exp-3 is incompletely separated in one female and one CV male (Fig. 9A). In another CV male, P1 exp-3 bears the two apical setae completely fused (not figured).

#### Etymology.

The new species, *changi*, is named after Professor Cheon Young Chang (Daegu University, Korea), who has contributed to the taxonomy of Korean meiofauna. It is a noun in the genitive case.

#### Remarks.

Among the known species of *Laophontella*, *L. changi* sp. nov. most closely resembles *L. horrida dentata* in having a backward projection on the first antennular segment, a fringe of long setules along the posteroventral margin of the genital double-somite, and several crenulate projections along the posteroventral margin of the third to fifth urosomites in males. However, the new species differs from *L. horrida dentata* in the segmentation of the female antennule (eight-segmented in *L. horrida dentata* vs. seven-segmented in *L. changi* sp. nov.), posteroventral margin of the second free abdominal somite in females (with toothlike projections in *L. horrida dentata* vs. smooth margin in *L. changi* sp. nov.), outer margin of the caudal rami of females (with two pointed processes in *L. horrida dentata* vs. C-shaped proximal dorsolateral expansion and auriform lateral elevation in *L. changi* sp. nov.), shape of the inner seta on the P2enp-2 of males (short and sickle-shaped in *L. horrida dentata* vs. long in *L. changi* sp. nov.), and shape of the modified outer element on the P3enp-2 of males (elongate in *L. horrida dentata* vs. hoe-shaped in *L. changi* sp. nov.).

Within the *L. horrida* complex, the new species differs from the two remaining congeners in the caudal rami of females: *L. horrida horrida* bears a strong spiniform lateral process, whereas *L. horrida namibiensis* possesses a laterally expanded and rounded outer margin. The new species also clearly differs from *L. armata armata*, which has a five-segmented antennule and cylindrical caudal rami in females (Table 1).

**Table 1.** Comparison of morphological characters among species of the genus *Laophontella*, including *L. changi* sp. nov.

Species	*L. typica*	*L. armata armata*	*L. armata indica*	*L. horrida horrida*		*L. horrida dentata*	*L. horrida namibiensis*	*L. changi* sp. nov.	
Stages in reference	CV ♂	Adult ♀♂	CV ♀ Adult ♂	Adult ♀	Adult ♀♂	Adult ♀♂	Adult ♀♂	CV ♀♂	Adult ♀♂
Body length ♀/♂ (mm)	– / 0.5	0.8 / –	0.74–0.78 / 0.76	1.2 / –	– / –	0.88–0.92 / 0.77–0.82	1.05 / 0.86	0.85–0.95 / 0.82–0.84	1.07–1.19 / 0.83–0.97
No. of antennular segments (♀/♂)	– / 5	5 / 6^*^	5 / 5^*^	8 / –	6 / 6	8 / –	6 / –	7 / 7	
Position of principal aesthetasc of ♀ antennule	–	1	2	3^*^	2^*^	3	2^*^	2	
No. of setae on basis of maxilliped	1	1^*^	1^*^	1^*^	–	2	1^*^	2	
Length-to-width ratio of caudal ramus (♀/♂)	– / 1.5^*^	5^*^ / 3^*^	3^*^ / 2.5^*^	1.7^*^ / –	0.8^*^ / 2^*^	1.4^*^ / 3^*^	1.4^*^ / 2.5^*^	1.1^*^ / 1.5^*^	1.6^*^ / 2^*^
Outer margin of ♀ caudal ramus	–	smooth^*^	smooth^*^	a strong spiniform lateral process	well-developed blunt-tipped projection^*^	two pointed processes^*^	circular	two protrusions consisting of C-shaped proximal dorsolateral expansion and auriform lateral elevation	
Armature formula of P4 endopod	020^*^	1.010	110	1.010^*^	1.010	1.010	1.010^*^	1.010	
Ratio of ♀ P4exp-2: inner seta of P4exp-2	–	1:3.6^* 1)^	1.6:1^*^	2.4:1^*^	1.2:1^*^	2.8:1^*^	1.7:1^*^	1:1.8^*^	1:1.5^*^
No. of setae in P4exp-3 (♀/♂)	– / 5^*^	5 / 5^*^	5 / 2	4 / –	5^*^ / –	5 / 5	4 / 4^*^	5 / 5	
No. of setae in ♂ P5exp	7	5–6	7	–	6	6	–	6 (exopod not separated)	6
Projection on ventrodistal edge of ♀ anal somite	–	absence^*^	absence^*^	presence (1 pair)	presence (1 pair)^*^	presence (2 pairs)	–	absence	
Reference	Thompson and Scott 1903	Willey 1935	Sewell 1940	Por 1964	Bodin 1964	Mielke 1992	Kunz 1994	This study	

Note. All data are based on original descriptions unless otherwise indicated. ^1)^ Cited from Geddes 1968 Symbols: –, unknown; ^*^, data measured from figures.

The type species, *L. typica*, and *L. armata indica* were originally described based on CV specimens (Wells and Rao 1987). *Laophontella typica* is interpreted as the CV male, as indicated by its nine-segmented body and non-foliaceous P5, whereas *L. armata indica* appears to represent the CV female, lacking a genital double-somite and a weakly foliaceous P5. The CV of *L. changi* sp. nov. differs from that of *L. typica* in lacking the additional outer projection on the first antennular segment, and from *L. armata indica* in possessing a different antennular segmentation (the latter having a five-segmented female antennule).

A comparative table was presented based on the characters for the recorded species of the genus *Laophontella* (Table 1).

## Discussion

Although the limits of the genus *Laophontella* are generally well defined by a stable combination of diagnostic characters, species-level delimitation remains challenging. As previously mentioned, the type species, *L. typica*, was established based on an incomplete description and illustrations of a sub-adult (CV stage) specimen, which has hindered interspecific comparisons. Consequently, the availability of both adult and CV stage specimens of the present new species provides a critical opportunity to evaluate morphological development. Accordingly, the following discussion focuses on the interpretation of diagnostic characters, specifically comparing the antennular segmentation, urosomal architecture, and caudal ramus structure between the adult and CV stage.

### Interpretation of morphological characters between adult and copepodid V stage

#### Antennule

Antennular segmentation is a key taxonomic character in harpacticoid copepods, yet it exhibits interspecific variation in the degree and pattern of segmental fusion (Huys and Boxshall 1991). In the family Tetragonicipitidae, the basic ground pattern of the female antennule is generally considered nine-segmented (Fiers 1995: Table II). However, members of *Laophontella* exhibit a range from five to eight segments.

In *L. changi* sp. nov., the antennular segmentation and setal formula in CV females are identical to those of the adult (Fig. 3A). The antennule in CV males similarly consists of seven segments; however, it differs from that of the adult male in its setation and in the lack of geniculation.

Copepodid development within the Tetragonicipitidae has been described for *Mwania phytocola* Fiers & de Troch, 2000 (CI–CV), and partially for *Neogoniceps martinezi* Fiers & de Troch, 2000 (CIV–CV). According to Fiers and de Troch (2000), the addition of segments first occurs distal to the principal aesthetasc-bearing segment (segment II) during the transition from CI to CII. The principal aesthetasc-bearing segment enlarges during the transition from CII to CIII, and subsequently undergoes subdivision during the transitions from CIII to CV, with the final adult number of segments being attained at the CV stage. In this context, the position of the principal aesthetasc-bearing segment is an important reference point and a useful character for distinguishing *Laophontella* species described from immature individuals, including the type species.

Accordingly, the species in which the principal aesthetasc-bearing segment of the female antennule corresponds to the second segment, as in the new species, include *L. armata indica* and *L. horrida namibiensis*. However, the female of the new species differs from the female of the other two species in having a seven-segmented antennule (vs. five-segmented in *L. armata indica*, and six-segmented in *L. horrida namibiensis*).

Although the developmental pattern of the male antennule cannot be clearly interpreted, comparison between the adult and the CV male of the new species shows that the antennule of the latter consists of seven segments, with the principal aesthetasc-bearing segment located on the second segment (vs. the fifth segment in the adult male), identical to the condition observed in the adult female. This indicates that the sexual dimorphism of the male antennule becomes apparent during the final molt from the CV to the adult stage. During this transition, the first segment of the CV male antennule bearing 12 setae, is presumed to be subdivided into the first and second segments of the adult male, bearing 1 and 11 setae, respectively. The distal three segments (fifth to seventh segments) of the CV male (with setal formula of 2, 2, 5 + acrothek) appear to be reorganized into the sixth and seventh segments of the adult male (with 1, 8 + acrothek), which suggests a combination of subdivision and fusion. It is presumed that the second segment of the CV male is partially subdivided into the third and fourth segments of the adult, while another portion fuses with third and fourth segments of the CV to form the fifth segment of the adult. This interpretation is supported by the apparent shift of the principal aesthetasc from the distal position on the second segment of the CV to a more central position on the fifth segment in the adult. Dahms (1989) reported a similar pattern in harpacticoid copepods during the transition from the CV to the adult stage, involving proximal segment addition and distal fusion, which can be inferred from the setal pattern and the position of aesthetasc-bearing segments.

#### Urosomites

In both CV females and males of the new species, the first and second free abdominal somites bear well-developed, paired dorsolateral projections and several crenulate projections along the posteroventral margin (Fig. 12F, G). These structures diverge in form as the organism reaches maturity. The projections seen in the CV are retained in the adult male, but become reduced and weakly developed (Figs 6F, 12E). In contrast, the adult female loses the crenulate projections along the posteroventral margin; this results in a ventral cuticular expansion with an undulating edge on the first free abdominal somite, and a comparatively smooth margin on the second free abdominal somite (Figs 2B, 12C).

This implies that abdominal projections are strongly expressed during the juvenile stage but undergo significant modification during the final development. A similar pattern has been reported in *L. horrida dentata*, in which the adult male retains crenulate projections on the first and second free abdominal somites (Mielke 1992: fig. 10C), whereas the adult female lacks these structures on the first free abdominal somite and retains only weak projections on the second (Mielke 1992: fig. 4B).

#### Caudal ramus

**Figure 13.**
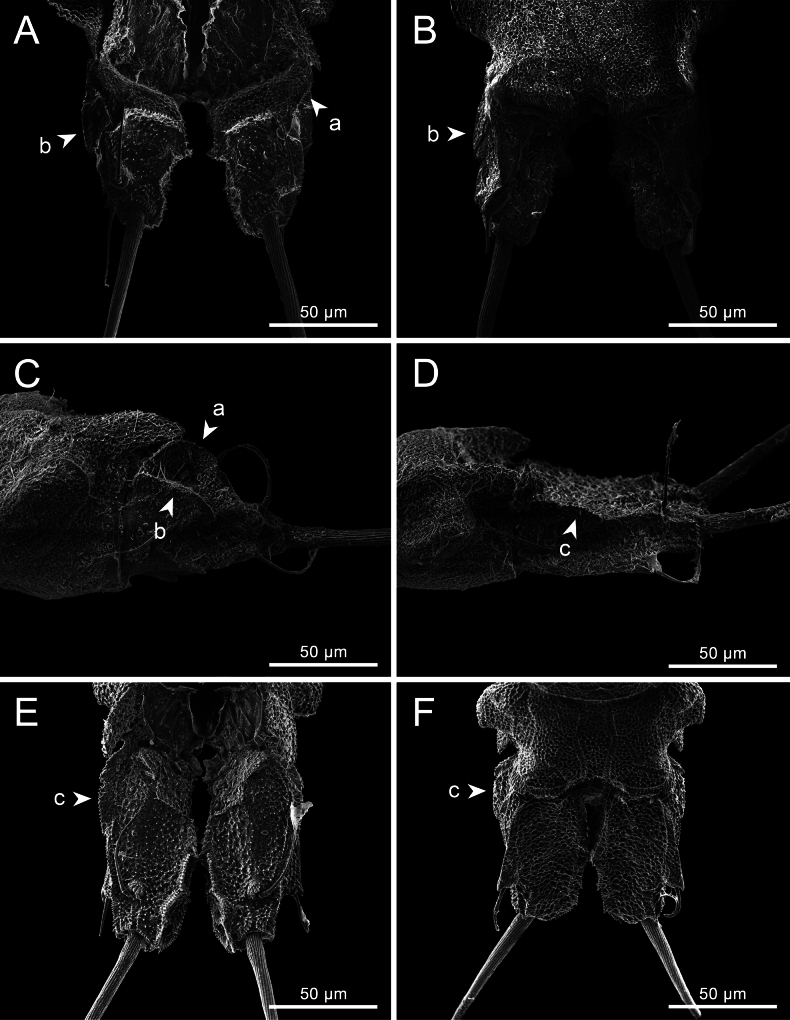
Scanning electron micrographs of *Laophontella changi* sp. nov., adult female. **A**. Caudal rami, dorsal; **B**. Same, ventral; **C**. Same, lateral. Adult male: **D**. Caudal rami, lateral; **E**. Same, dorsal; **F**. Same, ventral. **a, c**. A dorsolateral expansion; **b**. A lateral process.

**Figure 14.**
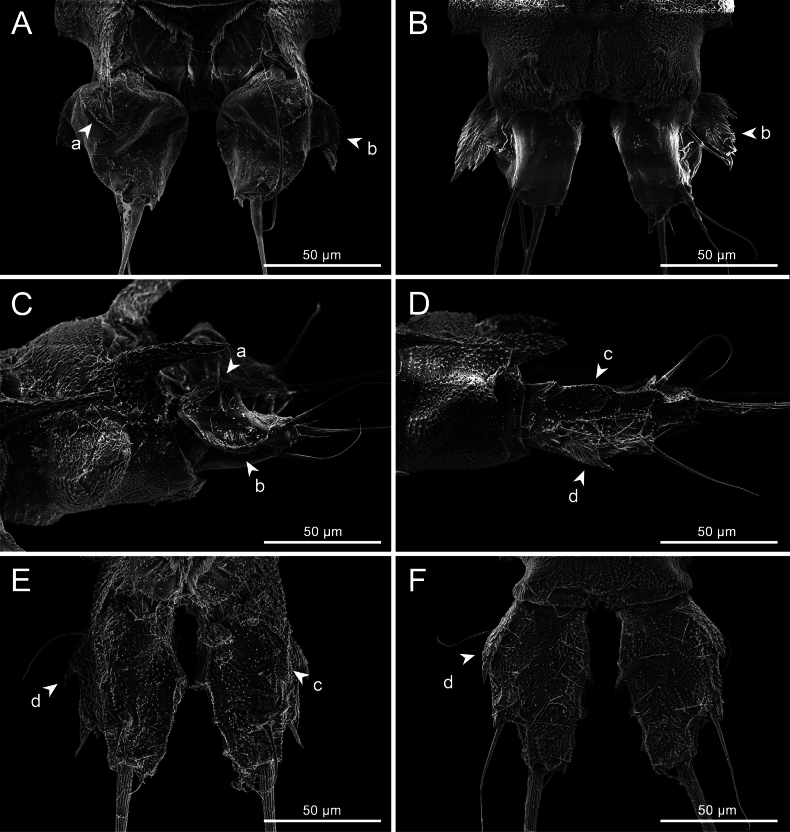
Scanning electron micrographs of *Laophontella changi* sp. nov., copepodid V female. **A**. Caudal rami, dorsal; **B**. Same, ventral; **C**. Same, lateral. Copepodid V male: **D**. Caudal rami, lateral; **E**. Same, dorsal; **F**. Same, ventral. **a, c**. A dorsolateral expansion; **b**. A lateral process; **d**. A ventrolateral process.

The transition from the CV to the adult stage in the new species involves an increase in the length-to-width ratio of the caudal ramus in both sexes, reflecting its elongation. In females, the ratio increases from 1.1 in the CV to 1.6 in the adult; in males, it increases from 1.5 to 2.0. Additionally, the size and shape of the cuticular outgrowths on the caudal ramus (‘a–d’ in Figs 7, 11, 13, 14) shift during development.

In CV females, the dorsolateral expansion (‘a’ in Figs 11A, 11B, 14A, 14C) located proximally on the caudal ramus is gently C-shaped in lateral view and weakly ornamented, but it becomes strongly ornamented in the adult (‘a’ in Figs 7A, 7B, 13A, 13C). The well-developed, wing-like lateral process (‘b’ in Figs 11A, 11B, 14A–C) is markedly reduced in the adult, forming a weakly arcuate elevation in dorsal view and an auriform elevation in lateral view (‘b’ in Figs 7A, 7B, 13A–C). In contrast, the dorsolateral expansion in CV males (‘c’ in Figs 11C, 11D, 14D, 14E) becomes more pronounced in the adult (‘c’ in Figs 7C, 7D, 13D, 13E), whereas the elongated ventrolateral process (‘d’ in Figs 11C, 11D, 14D–F) is reduced to a vestigial state in the adult (Figs 7C, 7D, 13D–F).

### Proposed characters for *Laophontella*

One notable feature of *L. changi* sp. nov. is the structure of the P1 coxa and basis. The P1 coxa of the new species is strongly expanded laterally; the basis arises from the inner margin of the coxa and occupies approximately one-quarter of its width (Fig. 4A). This condition is restricted to the P1. A similar structure is illustrated for *L. armata armata* sensu Geddes, 1968 (Geddes 1968: fig. 10A) and *L. horrida dentata* (Mielke 1992: fig. 7B), implying that this character is consistent within the genus *Laophontella*. Another notable feature is the fusion of the P4 intercoxal sclerite to the coxa in the new species (Figs 4D, 6C). This fusion is also shown in illustrations of *L. armata armata* of Willey (1935: fig. 145).

The P1 coxa in most other genera of the family Tetragonicipitidae is relatively small or weakly developed laterally, with most of its distal margin contacting the basis. Furthermore, the P4 intercoxal sclerite is typically separated from the coxa. However, an exception has been reported for *Oniscopsis* Chappuis, 1954, in which the P1 coxa is enlarged laterally and the P4 intercoxal sclerite is fused to the coxa (Kitazima 1983; Wells and Rao 1987). These observations support the previously proposed close relationship between *Laophontella* and *Oniscopsis* within Tetragonicipitidae (Kunz 1984), and may represent synapomorphies shared by the two genera. Although these characters may have been overlooked in early studies of *Laophontella*, the combination of a laterally expanded P1 coxa and the fusion of the P4 intercoxal sclerite may be useful for assessing relationships within the family.

## Supplementary Material

XML Treatment for Laophontella changi

## Additional information

### Artificial Intelligence (AI) use

The authors accept full responsibility for the content of the manuscript, including the disclosure of any use of AI.

No AI tools were used in the preparation of this manuscript.

### Author ORCIDs

J. Seo https://orcid.org/0009-0008-0912-9034

K. Cho https://orcid.org/0000-0002-5006-5713

J. Lee https://orcid.org/0000-0001-9004-8275

### Data availability

All of the data that support the findings of this study are available in the main text.
